# Phosphorylated Dihydroceramides from Common Human Bacteria Are Recovered in Human Tissues

**DOI:** 10.1371/journal.pone.0016771

**Published:** 2011-02-11

**Authors:** Frank C. Nichols, Xudong Yao, Bekim Bajrami, Julia Downes, Sydney M. Finegold, Erica Knee, James J. Gallagher, William J. Housley, Robert B. Clark

**Affiliations:** 1 Department of Oral Health and Diagnostic Sciences, University of Connecticut School of Dental Medicine, Farmington, Connecticut, United States of America; 2 Department of Chemistry, University of Connecticut, Storrs, Connecticut, United States of America; 3 Division of Infectious Diseases, VA Greater Los Angeles Healthcare System, Los Angeles, California, United States of America; 4 Department of Surgery, University of Connecticut School of Medicine, Farmington, Connecticut, United States of America; 5 Connecticut Vascular Institute, Hartford, Connecticut, United States of America; 6 Departments of Immunology and Medicine, School of Medicine, University of Connecticut, Farmington, Connecticut, United States of America; Institut de Pharmacologie et de Biologie Structurale, France

## Abstract

Novel phosphorylated dihydroceramide (PDHC) lipids produced by the periodontal pathogen *Porphyromonas gingivalis* include phosphoethanolamine (PE DHC) and phosphoglycerol dihydroceramides (PG DHC) lipids. These PDHC lipids mediate cellular effects through Toll-like receptor 2 (TLR2) including promotion of IL-6 secretion from dendritic cells and inhibition of osteoblast differentiation and function *in vitro* and *in vivo*. The PE DHC lipids also enhance (TLR2)-dependent murine experimental autoimmune encephalomyelitis (EAE), a model for multiple sclerosis. The unique non-mammalian structures of these lipids allows for their specific quantification in bacteria and human tissues using multiple reaction monitoring (MRM)-mass spectrometry (MS). Synthesis of these lipids by other common human bacteria and the presence of these lipids in human tissues have not yet been determined. We now report that synthesis of these lipids can be attributed to a small number of intestinal and oral organisms within the *Bacteroides*, *Parabacteroides*, *Prevotella*, *Tannerella* and *Porphyromonas* genera. Additionally, the PDHCs are not only present in gingival tissues, but are also present in human blood, vasculature tissues and brain. Finally, the distribution of these TLR2-activating lipids in human tissues varies with both the tissue site and disease status of the tissue suggesting a role for PDHCs in human disease.

## Introduction

*Porphyromonas gingivalis* is a periodontal pathogen strongly associated with development of destructive periodontal disease in adults. We have recently characterized the structures of novel phosphorylated dihydroceramide (PDHC) lipids produced by this organism [1]. PDHCs include both low mass (LM) and high mass (HM) forms of phosphoglycerol dihydroceramide (PG DHC) and phosphoethanolamine dihydroceramide (PE DHC) lipids. These lipids have unique non-mammalian structures [1] which allow for their specific quantification in bacteria and human tissues using multiple reaction monitoring (MRM)-mass spectrometry (MS). These PDHC lipids are important because of their capacity to engage Toll-like receptor 2 (TLR2) resulting in dendritic cell secretion of IL-6 [2] and inhibition of osteoblast differentiation and function [3]. In addition, we have shown that the PG DHC lipids markedly stimulate proinflammatory secretory responses in human gingival fibroblasts [1] and the PE DHC lipids enhance murine experimental autoimmune encephalomyelitis (EAE), a model for multiple sclerosis [2]. Others have shown that these lipids promote apoptosis in HUVEC cells in culture through activation of caspases 3, 6 and 9 [4]. In these previously reported studies, the PDHC lipid classes were isolated only from *P. gingivalis*. Little is known regarding the capacity of other common human bacteria to produce these lipids and most importantly, whether these lipids can be identified in human tissues distant from sites normally colonized by these bacteria. Therefore, the purpose of this investigation was to evaluate intestinal bacterial species as well as other periodontal organisms for their capacity to produce PDHCs and to examine blood and human tissue samples for the presence of these novel TLR2-activating bacterial lipids.

## Results

**Figure 1:**
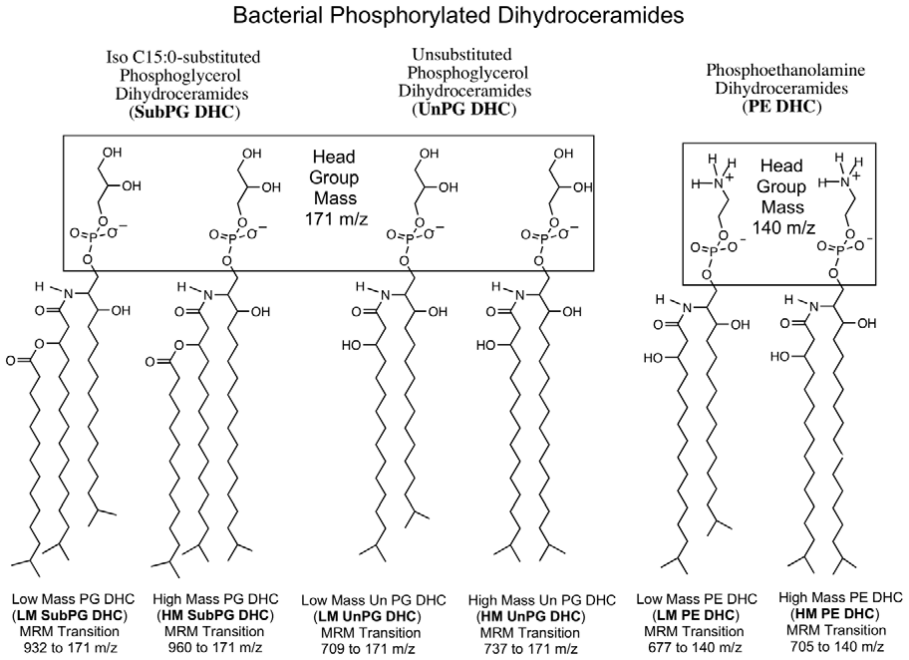
Structures of bacterial phosphorylated dihydroceramides (PDHC) lipids. Each lipid species was quantified using multiple reaction monitoring (MRM) mass spectrometry, via monitoring an MRM transition from the precursor lipid ion to the phosphorylated head group fragment ion generated upon collision-induced dissociation. The structures of low and high mass PDHC lipids are based on previously published reports [1], [5]. Though PG DHC and PE DHC lipids normally exist in high, medium and low masses, we elected to quantify only the high and low mass products because these vary most strongly between different bacterial species.

**Figure 2:**
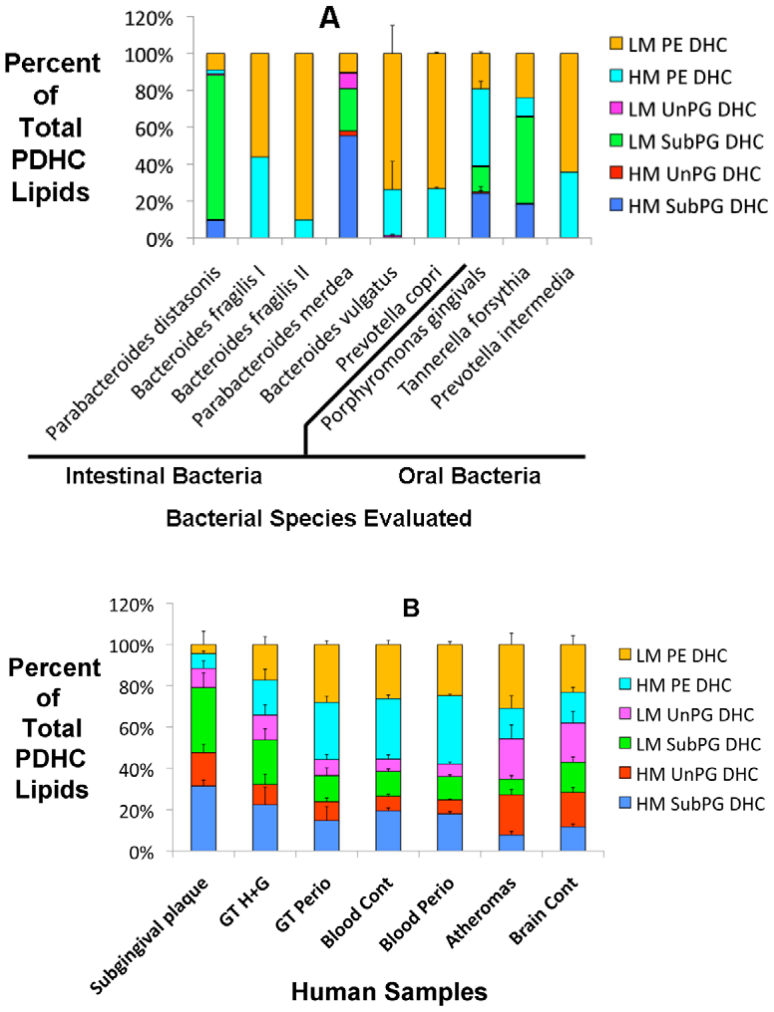
Recovery of bacterial phosphorylated dihydroceramides in intestinal and oral bacteria, subgingival plaque samples, blood plasma, atheroma and brain samples. Individual bacterial, blood and tissue samples were processed as described in the Methods section. The ion abundances of high and low mass PDHC lipid classes were summed and the recovery of each lipid class is depicted as the percent of the total ion abundance of the quantified PDHC lipids. Standard deviation bars are shown for lipid extracts from *Bacteroides vulgatus* (n = 13), *Prevotella copri* (n = 2), *Porphyromonas gingivalis* (n = 6), subgingival plaque (n = 2), healthy/mildly inflamed gingival tissue (GT H+G, n = 7), periodontitis gingival tissue samples (GT Perio, n = 6), control blood plasma (Blood Cont, n = 8), blood plasma from patients with generalized severe periodontitis (Blood Perio, n = 6), carotid atheroma (Atheroma, n = 11) and brain samples from deceased, neurologically-normal subjects (Brain Control, n = 14). Recovery of each lipid class is depicted as percent of the total PDHC ion recovery. Two-factor ANOVA indicated significant differences between categories of human samples. Comparison of PDHC lipid distributions in healthy/mildly inflamed versus periodontitis gingival tissue samples revealed significant differences for the percentage of HM SubPG DHC lipids, LM UnPG DHC lipids, LM PE DHC lipids.

We analyzed lipid extracts derived from greater than 240 individual human isolates representing over 90 intestinal bacterial species. Of the intestinal bacterial species evaluated, only approximately 5% produced PDHCs. Furthermore, only intestinal organisms of the *Bacteroides, Parabacteroides* or *Prevotella* genera produced PDHCs and of these, most produced predominantly PE DHC lipids (see Table S1 for the data set). Furthermore, the predominant PE DHC lipids produced by these intestinal organisms were usually the LM PE DHC form (see Figure 1 for lipid structures and Figure 2A for recovery of these lipids in intestinal and oral bacteria). Of the intestinal bacteria examined, only *Parabacteroides distasonis* and *Parabacteroides merdea* produced the PG DHC lipids, with *P. distasonis* producing primarily LM PG DHCs and *P. merdea* producing primarily HM PG DHCs (Figure 2A). We also examined lipid extracts from three bacterial species known to be associated with inflammatory periodontal disease in humans including *Porphyromonas gingivalis*, *Tannerella forsythia* and *Prevotella intermedia*. Figure 2A shows that these species vary in their capacity to produce either PE DHC or PG DHC lipids and also vary in their production of the HM versus the LM forms of these PDHCs. For example, the PDHC lipid constituents produced by *P. gingivalis* are predominantly HM PE DHC lipids whereas *T. forsythia* produces primarily LM PG DHC forms. Though individual intestinal and oral organisms vary in their capacity to produce specific PDHC lipids, combinations of these organisms also have the potential to deposit unique mixtures of PDHCs in human tissues and blood.

We next quantified bacterial PDHCs in human blood and tissue samples (see Table S2 for the data set). We examined blood plasma samples from periodontally healthy subjects, blood plasma samples from subjects with generalized severe destructive periodontal disease (chronic periodontitis), subgingival microbial plaque samples, and normal human brain samples. For carotid endarterectomy samples, we excised the patent segment of the common carotid artery (control samples) from the grossly apparent atheroma of the carotid body and quantified PDHCs within the lipid extracts of these paired carotid samples. The patent carotid artery samples showed no apparent gross atheroma formation though these artery segments usually were variably but significantly calcified within the artery wall.

We observed deposition of PDHCs in all of the human tissues examined, but the distribution of PDHCs in these tissues showed distinctive patterns. While the PDHCs derived directly from bacterial samples tended to contain primarily either HM or LM PDHCs, PDHC lipids in human tissue samples showed a mixture of HM and LM forms suggesting a heterogenous bacterial source (see Figure 2B). Nevertheless, tissue-associated patterns described below suggest that it may be possible to attribute a relatively specific distribution of bacterial PDHCs to various human tissues in health and in disease.

PG DHC lipids (both the HM and LM forms) can exist with three aliphatic chains (the substituted or “Sub” form) or can be de-esterified to a lipid class with 2 aliphatic chains (the unsubstituted or “Un” form) [1], [5]. We next compared the de-esterification status of the PG DHCs derived from bacteria with the PG DHCs derived from human tissue samples. Of the intestinal and periodontal organisms observed to produce PG DHCs, only *P. merdea* produced a small amount of the UnPG DHC lipids (<10% of total PDHC), whereas the remaining intestinal and oral bacteria produced negligible amounts of UnPG DHC lipids (Figure 2A).

**Figure 3:**
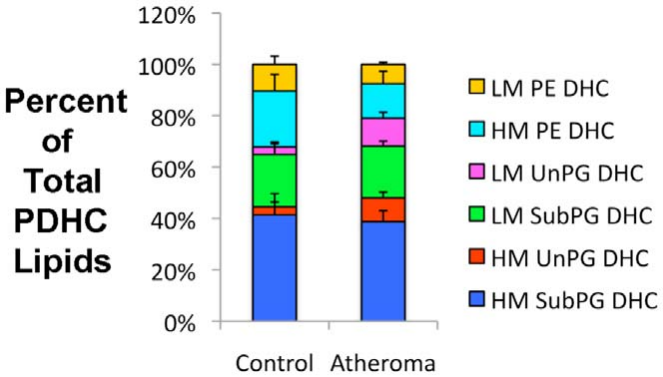
Recovery of bacterial phosphorylated dihydroceramides in paired patent artery and atheroma samples. For each carotid endarterectomy sample, the patent artery segment of the proximal common carotid artery was excised from the grossly evident atheroma located within the carotid sinus. The paired tissue samples were separately extracted for lipids as described in the Methods. A defined amount (approximately 3 µg of total lipids in 5 µl of HPLC solvent) of each lipid extract was analyzed by MRM-MS and the recovery of each lipid class is depicted as the percent of the total ion abundance of the quantified PDHC lipids. The percentages of PDHC lipids are depicted (means and standard errors) for five paired control and atheroma lipid extracts. Significant differences between control versus atheroma lipid extracts were shown only for the percentages of HM UnPG DHC and LM UnPG DHC lipids (p = 0.0144 and p = 0.0258, respectively, by paired t test). Though not shown, the mean abundances of PDHC ions per µg of total lipid extract were at least 33 times higher for the control artery samples compared with the atheroma samples.

In contrast, human tissue samples revealed significant percentages of both LM or HM UnPG DHC lipids (Figure 2B and Figure 3). We next examined blood plasma samples from periodontally healthy subjects and subjects with chronic periodontitis, and found that both revealed substantial percentages of both LM and HM UnPG DHC lipids (Figure 2B and Figure 3). In evaluating PG DHC de-esterification in atherosclerosis, we found that the lipid extracts from atheroma artery segments revealed significantly higher percentages of HM or LM UnPG DHC when compared with the control artery extracts. This suggests that atheroma formation is associated with greater de-esterification of deposited SubPG DHC lipids.

Of note, the average total ion abundances of PDHC lipids per microgram of total lipid extract were at least 33 times higher in the control artery segments than the atheroma segments (see Table S3 for the data set). In fact, the highest levels of PDHC lipids in lipid extracts from human tissue samples were observed in control artery segments, not gingival tissue samples from periodontal disease sites. The average levels of HM PE DHC and LM PE DHC lipids were at least 44 times higher in the control artery segments than the atheroma segments. This observation suggests that the vascular system proximal to (or upstream from) the site of atheroma formation is heavily contaminated with PDHC lipids. Since the control artery segments demonstrated variable degrees of mineralization based on the resistance to sectioning with a scalpel, the possibility exists that the elevated bacterial lipid accumulation is related to the process of mineralization in arterial walls without grossly evident atheroma development. The reduced levels of bacterial lipids in the atheroma lipid extracts compared to control carotid lipid extracts is probably accounted for by the overwhelming accumulation of cholesterol and cholesterol esters that are known to accompany atheroma formation. It is also possible that substantial cholesterol accumulation during atheroma formation could either prevent bacterial lipid accumulation in arterial walls or cause release of bacterial lipids from artery walls. These possibilities will be the subject of future research.

Lipid extracts of brain samples showed a mean percentage of UnPG DHC lipids comparable to or higher than those observed in carotid atheromas. In contrast, subgingival microbial plaque samples taken from gingival crevices at periodontitis sites showed only minimal levels of UnPG DHC. Subgingival plaque samples are known to contain microbial organisms as well as desquamated epithelial cells and inflammatory cells, and this combination apparently possesses the capability to partially de-esterify PG DHC lipids. These results suggest that metabolic conversion of the SubPG DHC lipids to the UnPG DHC lipids, while not occurring in the relevant bacteria, occurs both in blood and in some but not all human tissues. The percentage of UnPG DHC lipids in blood plasma samples compared with that in patent arterial samples and paired atheroma samples further suggests that the UnPG DHC lipids are not simply transported from blood to all tissues but may undergo specific metabolic conversion within certain tissues. Importantly, the biological properties of the SubPG DHC versus the UnPG DHC lipids remain to be evaluated.

We next compared the distribution of PDHC lipids in gingival tissue samples excised either from healthy/mildly inflamed or destructive periodontal disease (periodontitis) sites. We also evaluated blood plasma samples taken from periodontally healthy subjects or subjects with destructive periodontal disease. Two-factor ANOVA revealed significantly lower percentages of HM and LM SubPG DHC lipids and significantly higher percentages of HM and LM PE DHC lipids in periodontitis gingival tissue samples versus healthy samples (Figure 2B). Similarly, blood plasma samples demonstrated a significant increase in the percentage of HM PE DHC lipids in periodontitis plasma versus healthy plasma samples (though SubPG DHC percentages were not lower in plasma samples from periodontitis patients).

For quantification of absolute PDHC lipid levels in control and periodontitis gingival tissue samples, each tissue lipid extract was supplemented with 1.5 µg of synthetic phospholipid internal standard before analysis by MRM/MS. This analysis revealed that lipid extracts of periodontitis tissue samples contained significantly higher mean levels of HM PE DHC and LM PE DHC lipids than that recovered in healthy gingival tissue samples (p = 0.0062 and p = 0.0056, respectively, by one factor ANOVA, see Table S2). However, all other PDHC lipids were not significantly altered between periodontitis and healthy gingival tissue samples. Therefore, we now show that shifts in the deposition of specific bacterial lipids (PE DHCs) in gingival tissues is directly correlated with expression of destructive periodontal disease and that this specific increase in PE DHC is also reflected in blood plasma levels. Given that we have demonstrated that PE DHC can enhance murine autoimmunity [2], these results further suggest both a pro-inflammatory and tissue destructive role for PE DHC lipids and show that the absolute amount of PE DHC lipids in gingival tissues could be an important bacterial marker for expression of destructive periodontal disease in humans.

Overall, our findings indicate that human periodontal bacteria and a small percentage of human intestinal bacterial produce PDHC lipids, that these bacteria differ in the specific forms of PDHCs they produce, that PDHCs accumulate in numerous human tissues, and that the pattern of deposition varies not only among the tissue sites involved but also with the state of inflammation in those sites.

## Discussion

We have recently demonstrated that unique bacterially-derived lipids, termed PDHC lipids, promote cell activation through TLR2 [2], [3]. We now report that these bacterial lipids are recovered in human tissue samples and blood, making it plausible that PDHCs play a role in disease via acute and chronic activation of the immune system. The varying percentages of PDHC lipid species observed in either periodontal or intestinal bacterial species suggests that a mixture of bacterial species normally account for the overall blood and tissue deposition of these novel lipid classes. The fact that bacterial lipids are prevalent in patent artery segments suggests significant systemic exposure to these lipids. This may occur through release of PDHCs upon bacterial death, phagocytic engulfment of the PDHC-producing bacteria at their sites of origin, and/or through intermittent bacteremias. Recent studies have demonstrated that soluble bacterial peptidoglycan originating from the intestinal microflora can be recovered in the circulation and can subsequently prime neutrophils in bone marrow [6], [7]. Furthermore, ^13^C-labeled bacterial dihydroceramides ingested by experimental animals were shown to be distributed in skin, liver, skeletal muscle and brain, and a portion of the bacterial dihydroceramides were metabolized in liver to ceramides [8]. Therefore, our results demonstrating bacterial PDHC lipids in human tissues and de-esterification of SubPG DHC lipids in blood and tissues are consistent with these previous reports. That bacterial PDHC lipids are known to engage TLR2 in promoting cell activation of dendritic cells, macrophages or osteoblasts [2], [3] and are prevalent even in patent artery segments, raises the possibility that bacterial lipid deposition predisposes major arteries to the development of atherosclerosis. Furthermore, the relative predominance of PDHC lipids in control vessels suggests that bacterial lipid accumulation in the vasculature occurs before the majority of cholesterol and other mammalian lipids accumulate within atheromas. Whether blood and tissue PDHCs demonstrate transient variation in levels or fractional compositions and whether such variations correlate with inflammatory or other human diseases remains to be clarified.

We have demonstrated that PDHCs can promote autoimmune disease in mice and that this process is dependent on expression of TLR2 [2]. The role of the intestinal flora in both gastrointestinal and systemic immune activation has received considerable attention [9], [10], [11], [12], [13], [14]. We postulate that the intestinal and perhaps periodontal microbial production of PDHC lipids may also play a role in both the systemic immune homeostasis of the host and in disease mechanisms. For example, the relationship between severity of periodontal disease and rheumatoid arthritis [15], [16], [17], [18], [19] may relate to immune cell exposure to these lipids either systemically or through the direct deposition of these bacterial lipids in synovial sites.

Overall, our present findings provide a compelling rationale for future studies investigating the relationship between bacterial colonization of oral and intestinal sites, tissue deposition of PDHC lipids and the capacity of these uniquely structured, TLR2-activating lipids to promote human disease.

## Materials and Methods

For research involving human participants, written informed consent was obtained. The University of Connecticut Health Center Institutional Review Board specifically approved the human subject components of this study. Human atheroma, blood and plaque samples were stored frozen (−20°C) until the time of lipid extraction. Human brain specimens were obtained as postmortem samples from the Colorado Brain Bank, Denver, CO.

Intestinal bacterial samples that were previously stored frozen were grown on blood agar plates after demonstrating purity of bacterial isolates. The plates were scraped to recover the bacterial colonies and were extracted using the phospholipid extraction procedure of Bligh and Dyer [20] as modified by Garbus et al. [21]. *Porphyromonas gingivalis* (type strain, ATCC#33277), *Tannerella forsythia* (generously provided by Dr. Sigmund Socransky) and *Prevotella intermedia* (VPI 8944, generous gift of L.V. Moore) were grown in broth culture and after pelleting bacteria by centrifugation, the bacterial pellets were stored frozen until processing. At the time of lipid extraction, samples of bacterial pellets were removed and extracted using the same phospholipid extraction procedure.

All tissue and blood samples were stored frozen until processing. Gingival tissue, atheroma and brain samples were thawed and at least 20 mg of tissue was minced and extracted for several days in organic solvent according the method of Bligh and Dyer [20]. After drying organic solvent extracts under nitrogen, the lipid extracts were reconstituted in hexane∶isopropanol∶water (HPLC solvent, 6∶8∶0.75, v/v/v), vortexed and centrifuged. The resultant supernatants were recovered, a sample of defined volume (5µl) was dried and weighed, and a defined amount of each sample was transferred to a clean glass vial either for further processing or for MRM-MS analysis. For brain samples, 10 mg of each lipid extract was fractionated by normal phase HPLC as previously described [1] and the fractions expected to contain the PDHC lipids were pooled and dried. Each brain lipid isolate was then reconstituted in 300 µl of HPLC solvent and 5 µl was analyzed by MRM-MS for the bacterial lipids of interest. For each subgingival plaque sample, 50 µg of lipid extract was dissolved in 200 µl of HPLC solvent and 5 µl of each sample was analyzed by MRM-MS. For gingival tissue samples, each gingival lipid sample (1 mg) was supplemented with 50 µg of 1,2-di-(3,7,11,15-tetramethylhexadecanoyl)-sn-glycero-3-phosphoserine and the characteristic 847 to 311 m/z ion transition of this internal standard was used to correct for bacterial lipid levels between gingival tissue lipid samples. Each gingival lipid extract was then dissolved in 300 µl of HPLC solvent and 5 µl of each sample was analyzed by MRM-MS. Citrated blood samples, obtained by venipuncture from dental patients, were diluted 2∶1 in saline and subjected to Ficoll-Hypaque centrifugation. The recovered plasma samples were stored frozen until lipid extraction. For lipid extraction, the plasma samples were thawed and 0.5 ml of each sample was extracted for lipids as described above. The dried lipid samples were reconstituted in 300 µl of HPLC solvent and analyzed by MRM-MS.

Individual lipid samples were analyzed using a QTrap 4000 mass spectrometer (ABSciex). A standard volume of each lipid sample (5 µl) was analyzed by flow injection and HPLC solvent was run at a rate of 80 µl/min. Using previously purified lipid preparations of each phosphorylated dihydroceramide class, the instrument parameters were optimized for detection of each lipid component based on gas phase transitions depicted in Figure 1. The instrument parameters are listed in Table S4. Standard curves were generated using serially diluted lipid standards of known quantity and linearity of lipid quantification was observed (regression coefficients >0.98). In addition, carryover of individual lipid ion transitions into other monitored transitions was not observed. Using the optimized instrument parameters, each lipid extract from tissue, blood and bacterial samples was individually analyzed.

Each lipid ion transition peak was electronically integrated and the percentage abundance of each lipid class was calculated from the integrated lipid ion transition peaks. For each category of tissue or blood samples, all samples within a particular tissue or blood category were analyzed during a single analysis session. One or two-factor ANOVA, or the paired student t test was used to test for significance differences between sample categories.

## Supporting Information

Table S1 Ion abundances of bacterial phosphorylated dihydroceramides recovered from each intestinal and oral bacterial isolate. The individual bacterial samples were processed as described in the Materials and Methods and individual lipid extracts were evaluated by MRM-MS. Electronically integrated peaks depicted here for each bacterial isolate were used to generate the summary results shown in Figure 2A. Bacterial lipids that were not detected are listed as ND.
(DOC)

Table S2 Ion abundances of bacterial phosphorylated dihydroceramides recovered from individual lipid extracts of subgingival plaque samples, and human gingival tissue, blood, atheroma and brain samples. The individual tissue and blood specimens were processed as described in the Materials and Methods and individual lipid extracts were evaluated by MRM-MS. Electronically integrated peaks depicted here were used to generate the summary results shown in Figure 2B.
(DOC)

Table S3 Ion abundances of bacterial phosphorylated dihydroceramides in lipid extracts of paired common carotid (control) and carotid atheroma samples derived from human endarterectomy samples. The individual tissue specimens were processed as described in the Materials and Methods and individual lipid extracts were evaluated by MRM-MS. Electronically integrated peaks depicted here were used to generate the summary results shown in Figure 3.
(DOC)

Table S4 Mass spectrometric calibration parameters used to quantify bacterial lipids in bacterial or human specimens. The instrument parameters for the MRM-MS analysis are listed for the 4000QTrap Instrument (ABSciex). These parameters were defined using highly purified preparations of each PDHC lipid class.
(DOC)
