# Supplementary material for: Phosphorylated Dihydroceramides from Common Human Bacteria Are Recovered in Human Tissues
Source: PLoS One. 2011 Feb 11;6(2):e16771. doi: 10.1371/journal.pone.0016771 (PMC3037954; doi:10.1371/journal.pone.0016771)
Supplement: Table S1 — Ion abundances of bacterial phosphorylated dihydroceramides recovered from each intestinal and oral bacterial isolate. The individual bacterial samples were processed as described in the Materials and Methods and individual lipid extracts were evaluated by MRM-MS. Electronically integrated peaks depicted here for each bacterial isolate were used to generate the summary results shown in Figure 2A. Bacterial lipids that were not detected are listed as ND. (DOC) [file pone.0016771.s001.doc]

Table S1

|  |  |  |  |  | **PDHC Lipid Ion Abundances** | |  |  |
| --- | --- | --- | --- | --- | --- | --- | --- | --- |
| **Phylum** | **Intestinal Species** | Isolate Number | HM SubPG DHC | HM UnPG DHC | LM SubPG DHC | LM UnPG DHC | HM PE DHC | LM PE DHC |
| Actinobacteria | Bifidobacterium bifidum | 16292 | ND | ND | ND | ND | ND | ND |
| Actinobacteria | Bifidobacterium catenulatum 100% | 14668 | ND | ND | ND | ND | ND | ND |
| Actinobacteria | Bifidobacterium catenulatum/  pseudocatenulatum | 16291 | ND | ND | ND | ND | ND | ND |
| Actinobacteria | Bifidobacterium catenulatum/  pseudocatenulatum | 16309 | ND | ND | ND | ND | ND | ND |
| Actinobacteria | Bifidobacterium infantis | 14599 | ND | ND | ND | ND | ND | ND |
| Actinobacteria | Bifidobacterium infantis 100% | 14760 | ND | ND | ND | ND | ND | ND |
| Actinobacteria | Bifidobacterium pseudocatenulatum | 16101 | ND | ND | ND | ND | ND | ND |
| Actinobacteria | Bifidobacterium pseudocatenulatum | 16412 | ND | ND | ND | ND | ND | ND |
| Actinobacteria | Bifidobacterium pseudocatenulatum | 16433 | ND | ND | ND | ND | ND | ND |
| Actinobacteria | Bifidobacterium adolescentis 99.9% | 14695 | ND | ND | ND | ND | ND | ND |
| Actinobacteria | Collinsella aerofaciens | 16125 | ND | ND | ND | ND | ND | ND |
| Actinobacteria | Collinsella aerofaciens | 16390 | ND | ND | ND | ND | ND | ND |
| Actinobacteria | Coriobacterium sp | 16285 | ND | ND | ND | ND | ND | ND |
| Actinobacteria | Coriobacterium sp | 16285 | ND | ND | ND | ND | ND | ND |
| Actinobacteria | Coriobacterium sp | 16307 | ND | ND | ND | ND | ND | ND |
| Actinobacteria | Coriobacterium sp | 16331 | ND | ND | ND | ND | ND | ND |
| Actinobacteria | Coriobacterium sp | 16518 | ND | ND | ND | ND | ND | ND |
| Actinobacteria | Coriobacterium sp 100% | 14704 | ND | ND | ND | ND | ND | ND |
| Actinobacteria | Coriobacterium sp 100% | 16124 | ND | ND | ND | ND | ND | ND |
| Actinobacteria | Coriobacterium sp 100% | 16518 | ND | ND | ND | ND | ND | ND |
| Actinobacteria | Eggerthella biforme | 16378 | ND | ND | ND | ND | ND | ND |
| Actinobacteria | Eggerthella lenta | 16119 | ND | ND | ND | ND | ND | ND |
| Actinobacteria | Eggerthella lenta | 16453 | ND | ND | ND | ND | ND | ND |
| Actinobacteria | Eggerthella sp 99.3% | 16120 | ND | ND | ND | ND | ND | ND |
| Bacteroidetes | Alistipes onderdonkii | 16329 | ND | ND | ND | ND | ND | ND |
| Bacteroidetes | Bacteroides caccae (99%) | 14681 | ND | ND | ND | ND | ND | ND |
| Bacteroidetes | Bacteroides fragilis (***) | 14413 | ND | Trace | ND | Trace | 1447000 | 1860000 |
| Bacteroidetes | Bacteroides fragilis (12491) | 14377 | ND | ND | ND | ND | 1977000 | 18190000 |
| Bacteroidetes | Bacteroides ovatus | 16456 | ND | ND | ND | ND | ND | ND |
| Bacteroidetes | Bacteroides ovatus  (16s = 98%) | 14514 | 13920 | 15150 | 12180 | 15900 | 507000 | 5679000 |
| Bacteroidetes | Bacteroides sp 94% | 14692 | ND | ND | ND | ND | ND | ND |
| Bacteroidetes | Bacteroides sp. | 14589 | ND | 14390 | ND | 85670 | 192300 | 4667000 |
| Bacteroidetes | Bacteroides stercoris | 14684 | ND | 2197 | ND | 1610 | 608800 | 4358000 |
| Bacteroidetes | Bacteroides uniformis | 14585 | ND | ND | ND | ND | 1614000 | 9713000 |
| Bacteroidetes | Bacteroides uniformis | 14690 | ND | 13210 | ND | 3540 | 434300 | 1277000 |
| Bacteroidetes | Bacteroides vulgatus | 11547 | ND | 199900 | ND | 331300 | 20930000 | 64330000 |
| Bacteroidetes | Bacteroides vulgatus | 11596 | ND | 135600 | ND | 673500 | 13410000 | 67220000 |
| Bacteroidetes | Bacteroides vulgatus | 11828 | ND | 211400 | ND | 677400 | 12590000 | 40690000 |
| Bacteroidetes | Bacteroides vulgatus | 11852 | ND | 182200 | ND | 688200 | 192400000 | 71760000 |
| Bacteroidetes | Bacteroides vulgatus | 1187 | ND | 33780 | ND | 285200 | 8306000 | 37280000 |
| Bacteroidetes | Bacteroides vulgatus | 11909 | ND | 225300 | ND | 873700 | 15770000 | 61090000 |
| Bacteroidetes | Bacteroides vulgatus | 11980 | ND | 163000 | ND | 509000 | 17400000 | 49830000 |
| Bacteroidetes | Bacteroides vulgatus | 15464 | ND | 227500 | ND | 554000 | 20470000 | 47930000 |
| Bacteroidetes | Bacteroides vulgatus | 15987 | ND | 118700 | ND | 1119000 | 14400000 | 78200000 |
| Bacteroidetes | Bacteroides vulgatus | 16373 | ND | 102200 | ND | 739200 | 7204000 | 46900000 |
| Bacteroidetes | Bacteroides vulgatus | 16952 | ND | 274100 | ND | 720000 | 19420000 | 53390000 |
| Bacteroidetes | Bacteroides vulgatus | 17464 | ND | 226200 | ND | 826300 | 23190000 | 63330000 |
| Bacteroidetes | Bacteroides vulgatus | 14591 | ND | 33430 | ND | 56880 | 1127000 | 4984000 |
| Bacteroidetes | Bacteroides vulgatus | 14689 | ND | 31160 | ND | 69290 | 1820000 | 4999000 |
| Bacteroidetes | Bacteroides vulgatus | 16373 | 25060 | 129100 | 40830 | 1079000 | 6066000 | 41920000 |
| Bacteroidetes | Bacteroidetes | 14691 | ND | ND | ND | ND | ND | ND |
| Bacteroidetes | Parabacteroides distasonis | 14590 | 5900000 | 97760 | 46080000 | 422900 | 921900 | 5384000 |
| Bacteroidetes | Parabacteroides distasonis | 14590 | 117100 | 8609 | 957800 | 24070 | 20400 | 160900 |
| Bacteroidetes | Parabacteroides merdae (97%)(1253/1283) | 14372 | 6538000 | 297800 | 2700000 | 1013000 | 41340 | 1207000 |
| Bacteroidetes | Porphyromonas species (levii 89%) | 16413 | ND | ND | ND | ND | ND | ND |
| Bacteroidetes | Prevotella copri | 16317 | ND | ND | ND | ND | 1166000 | 3118000 |
| Bacteroidetes | Prevotella copri | 16317 | ND | ND | ND | ND | 747200 | 2092000 |
| Deltaproteobacteria | Bilophila wadsworthia | 783 | ND | ND | ND | ND | ND | ND |
| Deltaproteobacteria | Bilophila wadsworthia | 7959 | ND | ND | ND | ND | ND | ND |
| Deltaproteobacteria | Bilophila wadsworthia | 9077 | ND | ND | ND | ND | ND | ND |
| Deltaproteobacteria | Bilophila wadsworthia | 12415 | ND | ND | ND | ND | ND | ND |
| Deltaproteobacteria | Bilophila wadsworthia | 12608 | ND | ND | ND | ND | ND | ND |
| Deltaproteobacteria | Bilophila wadsworthia | 12615 | ND | ND | ND | ND | ND | ND |
| Deltaproteobacteria | Bilophila wadsworthia | 12656 | ND | ND | ND | ND | ND | ND |
| Deltaproteobacteria | Bilophila wadsworthia | 12663 | ND | ND | ND | ND | ND | ND |
| Deltaproteobacteria | Bilophila wadsworthia | 12675 | ND | ND | ND | ND | ND | ND |
| Deltaproteobacteria | Bilophila wadsworthia | 12981 | ND | ND | ND | ND | ND | ND |
| Deltaproteobacteria | Bilophila wadsworthia | 14016 | ND | ND | ND | ND | ND | ND |
| Deltaproteobacteria | Bilophila wadsworthia | 14126 | ND | ND | ND | ND | ND | ND |
| Deltaproteobacteria | Bilophila wadsworthia | 14345 | ND | ND | ND | ND | ND | ND |
| Deltaproteobacteria | Bilophila wadsworthia | 14493 | ND | ND | ND | ND | ND | ND |
| Deltaproteobacteria | Bilophila wadsworthia | 14636 | ND | ND | ND | ND | ND | ND |
| Deltaproteobacteria | Bilophila wadsworthia | 14646 | ND | ND | ND | ND | ND | ND |
| Deltaproteobacteria | Bilophila wadsworthia | 16272 | ND | ND | ND | ND | ND | ND |
| Deltaproteobacteria | Bilophila wadsworthia | 16793 | ND | ND | ND | ND | ND | ND |
| Deltaproteobacteria | Bilophila wadsworthia | 16873 | ND | ND | ND | ND | ND | ND |
| Deltaproteobacteria | Bilophila wadsworthia | 17467 | ND | ND | ND | ND | ND | ND |
| Deltaproteobacteria | Bilophila wadsworthia | 17752 | ND | ND | ND | ND | ND | ND |
| Firmicutes | Anaerofustis stercorihominis T.S. | 14563 | ND | ND | ND | ND | ND | ND |
| Firmicutes | Anaerotruncus colihominis | 16424 | ND | ND | ND | ND | ND | ND |
| Firmicutes | Anaerotruncus colihominis 99%(16424, 16429) | 14565 | ND | ND | ND | ND | ND | ND |
| Firmicutes | Anaerotruncus colihominis(100%) (14825) | 14766 | ND | ND | ND | ND | ND | ND |
| Firmicutes | Anaerotruncus colihominis(16093,16095,16097) | 16086 | ND | ND | ND | ND | ND | ND |
| Firmicutes | Blautia luti | 16432 | ND | ND | ND | ND | ND | ND |
| Firmicutes | Blautia luti | 16460 | ND | ND | ND | ND | ND | ND |
| Firmicutes | Blautia luti-like | 16380 | ND | ND | ND | ND | ND | ND |
| Firmicutes | Blautia producta | 16325 | ND | ND | ND | ND | ND | ND |
| Firmicutes | Clostridium species (91%) | 14538 | ND | ND | ND | ND | ND | ND |
| Firmicutes | Clostridium innocuum (98.8%) | 14710 | ND | ND | ND | ND | ND | ND |
| Firmicutes | Clostridium aminobutyricum | 16445 | ND | ND | ND | ND | ND | ND |
| Firmicutes | Clostridium bartletti | 16138 | ND | ND | ND | ND | ND | ND |
| Firmicutes | Clostridium bartletti | 16512 | ND | ND | ND | ND | ND | ND |
| Firmicutes | Clostridium bifermentans | 14414 | ND | ND | ND | ND | ND | ND |
| Firmicutes | Clostridium bifermentans | 16469 | ND | ND | ND | ND | ND | ND |
| Firmicutes | Clostridium bolteae (99.5%) | 14578 | ND | ND | ND | ND | ND | ND |
| Firmicutes | Clostridium bolteae 99.5%(16099) | 16090 | ND | ND | ND | ND | ND | ND |
| Firmicutes | Clostridium butyricum | 16094 | ND | ND | ND | ND | ND | ND |
| Firmicutes | Clostridium butyricum (100%) (16109,16118) | 16108 | ND | ND | ND | ND | ND | ND |
| Firmicutes | Clostridium disporicum | 16201 | ND | ND | ND | ND | ND | ND |
| Firmicutes | Clostridium disporicum | 16501 | ND | ND | ND | ND | ND | ND |
| Firmicutes | Clostridium disporicum 100% (774/774) | 14701 | ND | ND | ND | ND | ND | ND |
| Firmicutes | Clostridium glycolicum | 16384 | ND | ND | ND | ND | ND | ND |
| Firmicutes | Clostridium glycolicum | 16465 | ND | ND | ND | ND | ND | ND |
| Firmicutes | Clostridium hypermegas | 16397 | ND | ND | ND | ND | ND | ND |
| Firmicutes | Clostridium innocuum (14404) | 14403 | ND | ND | ND | ND | ND | ND |
| Firmicutes | Clostridium lactifermentans | 16216 | ND | ND | ND | ND | ND | ND |
| Firmicutes | Clostridium orbiscindens | 16210 | ND | ND | ND | ND | ND | ND |
| Firmicutes | Clostridium orbiscindens | 16290 | ND | ND | ND | ND | ND | ND |
| Firmicutes | Clostridium orbiscindens | 16343 | ND | ND | ND | ND | ND | ND |
| Firmicutes | Clostridium orbiscindens | 16352 | ND | ND | ND | ND | ND | ND |
| Firmicutes | Clostridium orbiscindens | 16377 | ND | ND | ND | ND | ND | ND |
| Firmicutes | Clostridium orbiscindens | 16406 | ND | ND | ND | ND | ND | ND |
| Firmicutes | Clostridium orbiscindens | 16444 | ND | ND | ND | ND | ND | ND |
| Firmicutes | Clostridium orbiscindens | 16510 | ND | ND | ND | ND | ND | ND |
| Firmicutes | Clostridium orbiscindens (100%) (14580, 17455,17456) | 14567 | ND | ND | ND | ND | ND | ND |
| Firmicutes | Clostridium orbiscindens (14384) | 14383 | ND | ND | ND | ND | ND | ND |
| Firmicutes | Clostridium orbiscindens (16088,16089,16096,16100,16107) | 16087 | ND | ND | ND | ND | ND | ND |
| Firmicutes | Clostridium orbiscindens (97.9%)(14672, 14709) | 14671 | ND | ND | ND | ND | ND | ND |
| Firmicutes | Clostridium paraputrificum | 14502 | ND | ND | ND | ND | ND | ND |
| Firmicutes | Clostridium paraputrificum | 16298 | ND | ND | ND | ND | ND | ND |
| Firmicutes | Clostridium paraputrificum | 16480 | ND | ND | ND | ND | ND | ND |
| Firmicutes | Clostridium paraputrificum | 16514 | ND | ND | ND | ND | ND | ND |
| Firmicutes | Clostridium paraputrificum (14364, 14369) | 14416 | ND | ND | ND | ND | ND | ND |
| Firmicutes | Clostridium paraputrificum (14369,14416) | 14364 | ND | ND | ND | ND | ND | ND |
| Firmicutes | Clostridium paraputrificum (14661,14708) | 14660 | ND | ND | ND | ND | ND | ND |
| Firmicutes | Clostridium perfringens | 16110 | ND | Trace | ND | Trace | ND | ND |
| Firmicutes | Clostridium perfringens | 16202 | ND | ND | ND | ND | ND | ND |
| Firmicutes | Clostridium perfringens | 16203 | ND | Trace | ND | Trace | ND | ND |
| Firmicutes | Clostridium perfringens | 16299 | ND | Trace | ND | Trace | ND | ND |
| Firmicutes | Clostridium perfringens | 16302 | ND | ND | ND | ND | ND | ND |
| Firmicutes | Clostridium perfringens | 16304 | ND | ND | ND | ND | ND | ND |
| Firmicutes | Clostridium perfringens | 16448 | ND | ND | ND | ND | ND | ND |
| Firmicutes | Clostridium perfringens | 16479 | ND | ND | ND | ND | ND | ND |
| Firmicutes | Clostridium perfringens | 16497 | Trace | Trace | Trace | Trace | ND | ND |
| Firmicutes | Clostridium perfringens | 14572 | ND | ND | ND | ND | ND | ND |
| Firmicutes | Clostridium perfringens (14363) | 14417 | ND | ND | ND | ND | ND | ND |
| Firmicutes | Clostridium perfringens (14417) | 14363 | ND | ND | ND | ND | ND | ND |
| Firmicutes | Clostridium perfringens (14659) | 14658 | ND | ND | ND | ND | ND | ND |
| Firmicutes | Clostridium ramosum | 16356 | ND | ND | ND | ND | ND | ND |
| Firmicutes | Clostridium ramosum(14609) | 14600 | ND | ND | ND | ND | ND | ND |
| Firmicutes | Clostridium rectum | 16486 | ND | ND | ND | ND | ND | ND |
| Firmicutes | Clostridium roseum 99.6% | 16085 | ND | ND | ND | ND | ND | ND |
| Firmicutes | Clostridium scindens (99.9%)(14528) | 14527 | ND | ND | ND | ND | ND | ND |
| Firmicutes | Clostridium scindens (99.8%)(see 17447) | 14566 | ND | ND | ND | ND | ND | ND |
| Firmicutes | Clostridium sordellii | 16482 | ND | ND | ND | ND | ND | ND |
| Firmicutes | Clostridium spiroforme | 16481 | ND | ND | ND | ND | ND | ND |
| Firmicutes | Clostridium subterminale | 16455 | ND | ND | ND | ND | ND | ND |
| Firmicutes | Clostridium subterminale | 16483 | ND | ND | ND | ND | ND | ND |
| Firmicutes | Clostridium subterminale | 16499 | ND | ND | ND | ND | ND | ND |
| Firmicutes | Clostridium subterminale | 14415 | ND | ND | ND | ND | ND | ND |
| Firmicutes | Clostridium subterminale | 14662 | ND | ND | ND | ND | ND | ND |
| Firmicutes | Clostridium symbiosum | 14673 | ND | ND | ND | ND | ND | ND |
| Firmicutes | Clostridium tertium | 16200 | ND | ND | ND | ND | ND | ND |
| Firmicutes | Coprobacillus cateniformis | 14501 | ND | ND | ND | ND | ND | ND |
| Firmicutes | Coprobacillus cateniformis | 16463 | ND | ND | ND | ND | ND | ND |
| Firmicutes | Eub tenue/C. sordelli | 16197 | ND | ND | ND | ND | ND | ND |
| Firmicutes | Eub tenue/C. sordelli-16S by Yuli | 16423 | ND | ND | ND | ND | ND | ND |
| Firmicutes | Eubacterium aerofaciens | 16340 | ND | ND | ND | ND | ND | ND |
| Firmicutes | Eubacterium formicigenerans (94.5%) | 16431 | ND | ND | ND | ND | ND | ND |
| Firmicutes | Eubacterium sp | 16454 | ND | ND | ND | ND | ND | ND |
| Firmicutes | Eubacterium sp (95.2%)(14577) | 14571 | ND | ND | ND | ND | ND | ND |
| Firmicutes | Eubacterium sp. III-35 99% | 14757 | ND | ND | ND | ND | ND | ND |
| Firmicutes | Eubacterium sp. III-35 99% | 14758 | ND | ND | ND | ND | ND | ND |
| Firmicutes | Finegoldia magna | 14533 | ND | ND | ND | ND | ND | ND |
| Firmicutes | Finegoldia magna | 14677 | ND | ND | ND | ND | ND | ND |
| Firmicutes | Finegoldia magna | 16126 | ND | Trace | ND | Trace | ND | ND |
| Firmicutes | Finegoldia magna | 16366 | ND | ND | ND | ND | ND | ND |
| Firmicutes | Finegoldia magna-17(99.6%) | 14719 | ND | ND | ND | ND | ND | ND |
| Firmicutes | Fusobacterium prausnitzii 88% | 14539 | ND | ND | ND | ND | ND | ND |
| Firmicutes | Group 23 | 14670 | ND | ND | ND | ND | ND | ND |
| Firmicutes | Group 23 | 16419 | ND | ND | ND | ND | ND | ND |
| Firmicutes | Group 23 | 16508 | ND | ND | ND | ND | ND | ND |
| Firmicutes | Holdemania filiformis | 14586 | ND | ND | ND | ND | ND | ND |
| Firmicutes | Holdemania filiformis | 14714 | ND | ND | ND | ND | ND | ND |
| Firmicutes | Peptoniphilus harei-2 | 14699 | ND | ND | ND | ND | ND | ND |
| Firmicutes | Parvimonas micra-15 (100%) | 14554 | ND | ND | ND | ND | ND | ND |
| Firmicutes | Parvimonas micra-16 (100%) | 14555 | ND | ND | ND | ND | ND | ND |
| Firmicutes | Parvimonas micra-18 (100%) | 14707 | ND | ND | ND | ND | ND | ND |
| Firmicutes | Peptostreptococcus sp CCUG42997-1 (99.9%) | 14718 | ND | ND | ND | ND | ND | ND |
| Firmicutes | Peptostreptococcus sp. oral clone CK035 | 14698 | ND | ND | ND | ND | ND | ND |
| Firmicutes | Ruminococcus -like (14783, 14784, 14788, 14789) | 14773 | ND | ND | ND | ND | ND | ND |
| Firmicutes | Ruminococcus | 16386 | ND | ND | ND | ND | ND | ND |
| Firmicutes | Ruminococcus flavefaciens | 16399 | ND | ND | ND | ND | ND | ND |
| Firmicutes | Ruminococcus gnavus | 14423 | ND | ND | ND | ND | ND | ND |
| Firmicutes | Ruminococcus gnavus | 16114 | ND | ND | ND | ND | ND | ND |
| Firmicutes | Ruminococcus gnavus | 16132 | ND | ND | ND | ND | ND | ND |
| Firmicutes | Ruminococcus gnavus | 16349 | ND | ND | ND | ND | ND | ND |
| Firmicutes | Ruminococcus gnavus | 16401 | ND | ND | ND | ND | ND | ND |
| Firmicutes | Ruminococcus gnavus | 16420 | ND | ND | ND | ND | ND | ND |
| Firmicutes | Ruminococcus gnavus | 16441 | ND | ND | ND | ND | ND | ND |
| Firmicutes | Ruminococcus gnavus (14576, 16420) | 14570 | ND | ND | ND | ND | ND | ND |
| Firmicutes | Ruminococcus gnavus (99.5%) | 16098 | ND | ND | ND | ND | ND | ND |
| Firmicutes | Ruminococcus group 23 | 14368 | ND | ND | ND | ND | ND | ND |
| Firmicutes | Ruminococcus group 23 | 16121 | ND | ND | ND | ND | ND | ND |
| Firmicutes | Ruminococcus group 23 | 16395 | ND | ND | ND | ND | ND | ND |
| Firmicutes | Ruminococcus lactaris 99.3% | 14694 | ND | ND | ND | ND | ND | ND |
| Firmicutes | Ruminococcus lactaris 99.3% | 14759 | ND | ND | ND | ND | ND | ND |
| Firmicutes | Ruminococcus obeum | 16385 | ND | ND | ND | ND | ND | ND |
| Firmicutes | Ruminococcus obeum (97%) | 14366 | ND | ND | ND | ND | ND | ND |
| Firmicutes | Ruminococcus obeum 100% | 14795 | ND | ND | ND | ND | ND | ND |
| Firmicutes | Ruminococcus species group 23 | 16201 | ND | ND | ND | ND | ND | ND |
| Firmicutes | Sarcina ventriculi | 16371 | ND | ND | ND | ND | ND | ND |
| Firmicutes | Sarcina ventriculi | 16103 | ND | ND | ND | ND | ND | ND |
| Firmicutes | Uncultured rumen clone 85% Ab034070 | 14542 | ND | ND | ND | ND | ND | ND |
| Firmicutes | Veillonella atypica--but this was B ovatus phenotypically! | 14446 | ND | ND | ND | ND | ND | ND |
| Firmicutes | Veillonella dispar (100%) | 14725 | ND | ND | ND | ND | ND | ND |
| Firmicutes | Veillonella sp 98.8% | 14693 | ND | ND | ND | ND | ND | ND |
| Fusobacteria | Catenibacterium mitsuokai | 16315 | ND | ND | ND | ND | ND | ND |
| Fusobacteria | Cetobacterium somerae | 14409 | ND | ND | ND | ND | ND | ND |
| Proteobacteria | Desulfovibrio | 7757 | ND | ND | ND | ND | ND | ND |
| Proteobacteria | Desulfovibrio | 8236 | ND | ND | ND | ND | ND | ND |
| Proteobacteria | Desulfovibrio | 8381 | ND | ND | ND | ND | ND | ND |
| Proteobacteria | Desulfovibrio | 8907 | ND | ND | ND | ND | ND | ND |
| Proteobacteria | Desulfovibrio | 8933 | ND | ND | ND | ND | ND | ND |
| Proteobacteria | Desulfovibrio | 8951 | ND | ND | ND | ND | ND | ND |
| Proteobacteria | Desulfovibrio | 9070 | ND | ND | ND | ND | ND | ND |
| Proteobacteria | Desulfovibrio | 9120 | ND | ND | ND | ND | ND | ND |
| Proteobacteria | Desulfovibrio | 9706 | ND | ND | ND | ND | ND | ND |
| Proteobacteria | Desulfovibrio | 9976 | ND | ND | ND | ND | ND | ND |
| Proteobacteria | Desulfovibrio | 10831 | ND | ND | ND | ND | ND | ND |
| Proteobacteria | Desulfovibrio | 11081 | ND | ND | ND | ND | ND | ND |
| Proteobacteria | Desulfovibrio | 11094 | ND | ND | ND | ND | ND | ND |
| Proteobacteria | Desulfovibrio | 11232 | ND | ND | ND | ND | ND | ND |
| Proteobacteria | Desulfovibrio | 11346 | ND | ND | ND | ND | ND | ND |
| Proteobacteria | Desulfovibrio | 11378 | ND | ND | ND | ND | ND | ND |
| Proteobacteria | Desulfovibrio | 11437 | ND | ND | ND | ND | ND | ND |
| Proteobacteria | Desulfovibrio | 11568 | ND | ND | ND | ND | ND | ND |
| Proteobacteria | Desulfovibrio | 11623 | ND | ND | ND | ND | ND | ND |
| Proteobacteria | Desulfovibrio | 11717 | ND | ND | ND | ND | ND | ND |
| Proteobacteria | Desulfovibrio | 11789 | ND | ND | ND | ND | ND | ND |
| Proteobacteria | Desulfovibrio | 11830 | ND | ND | ND | ND | ND | ND |
| Proteobacteria | Desulfovibrio | 11949 | ND | ND | ND | ND | ND | ND |
| Proteobacteria | Desulfovibrio | 13966 | ND | ND | ND | ND | ND | ND |
| Proteobacteria | Desulfovibrio | 29098 | ND | ND | ND | ND | ND | ND |
| Unknown | Butyrate-producing bacteria | 16462 | ND | ND | ND | ND | ND | ND |
| Unknown | Butyrate-producing bacterium 99%, P Lawson also (Clos sp 14505)99% | 14505 | ND | ND | ND | ND | ND | ND |
| Unknown | Novel (Ruminococcus-like) | 16519 | ND | ND | ND | ND | ND | ND |
| Unknown | Novel species | 16092 | ND | ND | ND | ND | ND | ND |
| Unknown | Novel species | 16106 | ND | ND | ND | ND | ND | ND |
| Unknown | Novel species | 16135 | ND | ND | ND | ND | ND | ND |
| Unknown | Novel species | 16332 | ND | ND | ND | ND | 8198000 | 156600000 |
| Unknown | Novel species | 16442 | ND | ND | ND | ND | ND | ND |
| Unknown | Novel species (Clostridium-like) | 14774 | ND | ND | ND | ND | ND | ND |
|  |  |  |  |  |  |  |  |  |
| **Phylum** | **Oral Species** |  |  |  |  |  |  |  |
| Bacteroidetes | Porphyrmonas gingivalis ATCC 33277 | Sample 1 | 18830000 | 505700 | 8748000 | 189700 | 33470000 | 13930000 |
| Bacteroidetes | Porphyrmonas gingivalis ATCC 33277 | Sample 2 | 7183000 | 299400 | 3267000 | 131300 | 15080000 | 6059000 |
| Bacteroidetes | Porphyrmonas gingivalis ATCC 33277 | Sample 3 | 11240000 | 398200 | 5035000 | 161500 | 23450000 | 8961000 |
| Bacteroidetes | Porphyrmonas gingivalis ATCC 33277 | Sample 4 | 22060000 | 644000 | 13690000 | 247000 | 36140000 | 18370000 |
| Bacteroidetes | Porphyrmonas gingivalis ATCC 33277 | Sample 5 | 24600000 | 671000 | 15180000 | 255700 | 36430000 | 18240000 |
| Bacteroidetes | Porphyrmonas gingivalis ATCC 33277 | Sample 6 | 14440000 | 509900 | 9852000 | 208800 | 24950000 | 12480000 |
| Bacteroidetes | Prevotella intermedia VPI 8944 |  | ND | 17850 | ND | 18650 | 17940000 | 32300000 |
| Bacteroidetes | Tannerella forsythia |  | ND | 32230 | ND | 83050 | 4287000 | 10300000 |
