# Supplementary material for: Phosphorylated Dihydroceramides from Common Human Bacteria Are Recovered in Human Tissues
Source: PLoS One. 2011 Feb 11;6(2):e16771. doi: 10.1371/journal.pone.0016771 (PMC3037954; doi:10.1371/journal.pone.0016771)
Supplement: Table S2 — Ion abundances of bacterial phosphorylated dihydroceramides recovered from individual lipid extracts of subgingival plaque samples, and human gingival tissue, blood, atheroma and brain samples. The individual tissue and blood specimens were processed as described in the Materials and Methods and individual lipid extracts were evaluated by MRM-MS. Electronically integrated peaks depicted here were used to generate the summary results shown in Figure 2B. (DOC) [file pone.0016771.s002.doc]

Table S2

|  |  |  | PDHC Lipid Ion Abundances | |  |  |
| --- | --- | --- | --- | --- | --- | --- |
| Sample Type | HM SubPG DHC | HM UnPG DHC | LM SubPG DHC | LM UnPG DHC | HM PE DHC | LM PE DHC |
| Subgingival Plaque #1 | 200700 | 78550 | 219600 | 52470 | 26940 | 20870 |
| Subgingival Plaque #2 | 63740 | 40800 | 56950 | 21200 | 21380 | 11630 |
| Analyzed 1.25 ug of each plaque lipid sample. |  |  |  |  |  |  |
| Gingival tissue samples (GT) |  |  |  |  |  |  |
| GT Healthy/Gingivitis #1 | 5514 | 0 | 4285 | 0 | 2721 | 730 |
| GT Healthy/Gingivitis #2 | 5315 | 4247 | 4755 | 4379 | 4625 | 5103 |
| GT Healthy/Gingivitis #3 | 5165 | 3173 | 5236 | 3660 | 2912 | 4375 |
| GT Healthy/Gingivitis #4 | 6481 | 3415 | 6689 | 3896 | 6439 | 6695 |
| GT Healthy/Gingivitis #5 | 5414 | 2229 | 4919 | 2987 | 6003 | 5395 |
| GT Healthy/Gingivitis #6 | 4588 | 2517 | 5508 | 3770 | 1926 | 4411 |
| GT Healthy/Gingivitis #7 | 5420 | 3922 | 5801 | 5064 | 6842 | 6879 |
|  |  |  |  |  |  |  |
| GT Periodontitis #1 | 4537 | 3800 | 4133 | 4442 | 12880 | 10890 |
| GT Periodontitis #2 | 26550 | 5457 | 10540 | 5366 | 24920 | 22130 |
| GT Periodontitis #3 | 10230 | 7795 | 9700 | 6983 | 23010 | 21380 |
| GT Periodontitis #4 | 6922 | 4026 | 5100 | 4282 | 12250 | 13620 |
| GT Periodontitis #5 | 6494 | 6771 | 7988 | 7076 | 19670 | 22170 |
| GT Periodontitis #6 | 9776 | 8863 | 15760 | 1061 | 19440 | 24210 |
| Analyzed 16.67 ug of each gingival tissue lipid extract. |  |  |  |  |  |  |
| Blood Control #1 | 60260 | 22550 | 39200 | 21850 | 70940 | 65650 |
| Blood Control #2 | 63040 | 21180 | 39780 | 18290 | 81350 | 76050 |
| Blood Control #3 | 66990 | 19660 | 40480 | 18370 | 103200 | 93690 |
| Blood Control #4 | 70320 | 21480 | 37870 | 17810 | 101200 | 100300 |
| Blood Control #5 | 60230 | 19580 | 39650 | 18010 | 94320 | 78770 |
| Blood Control #6 | 64700 | 29290 | 38190 | 17050 | 115200 | 103200 |
| Blood Control #7 | 60870 | 25070 | 37300 | 22510 | 105700 | 87100 |
| Blood Control #8 | 45880 | 20560 | 28770 | 18030 | 70480 | 61300 |
|  |  |  |  |  |  |  |
| Blood Perio #1 | 57780 | 18080 | 36120 | 9516 | 96990 | 76000 |
| Blood Perio #2 | 75240 | 25990 | 46850 | 24420 | 125600 | 94600 |
| Blood Perio #3 | 59280 | 22230 | 37560 | 22370 | 108500 | 79270 |
| Blood Perio #4 | 53160 | 21080 | 35450 | 19380 | 101600 | 73960 |
| Blood Perio #5 | 66890 | 30250 | 43460 | 31420 | 131600 | 101200 |
| Blood Perio #6 | 84230 | 33330 | 49740 | 23170 | 168800 | 123200 |
| See Materials and Methods for processing of blood samples. |  |  |  |  |  |  |
| Carotid Atheroma #1 | 4612 | 11690 | 4445 | 13180 | 3898 | 20080 |
| Carotid Atheroma #2 | 39160 | 57620 | 38930 | 78070 | 52310 | 116700 |
| Carotid Atheroma #3 | 21140 | 39250 | 21270 | 59690 | 35320 | 101300 |
| Carotid Atheroma #4 | 14430 | 29320 | 15040 | 39060 | 38790 | 67470 |
| Carotid Atheroma #5 | 27190 | 40880 | 27510 | 73330 | 43780 | 121800 |
| Carotid Atheroma #6 | 11670 | 28750 | 12200 | 35710 | 17960 | 63430 |
| Carotid Atheroma #7 | 21250 | 38730 | 20870 | 56040 | 37790 | 83760 |
| Carotid Atheroma #8 | 15400 | 19510 | 13780 | 26480 | 46700 | 48010 |
| Carotid Atheroma #9 | 19730 | 35280 | 15450 | 46480 | 34610 | 86310 |
| Carotid Atheroma #10 | 30340 | 72040 | 33130 | 94490 | 46220 | 101500 |
| Carotid Atheroma #11 | 33550 | 52990 | 32820 | 72120 | 102800 | 120800 |
| Analyzed 12.5 ug of each atheroma lipid extract. |  |  |  |  |  |  |
| Brain Sample #1 | 155900 | 224700 | 181100 | 257900 | 174000 | 281900 |
| Brain Sample #2 | 151799 | 245000 | 170700 | 273500 | 165700 | 256300 |
| Brain Sample #3 | 114100 | 176100 | 128600 | 199200 | 155300 | 263300 |
| Brain Sample #4 | 96330 | 157500 | 107900 | 171300 | 112700 | 172500 |
| Brain Sample #5 | 147800 | 198100 | 154700 | 229400 | 139800 | 243000 |
| Brain Sample #6 | 152600 | 225300 | 167800 | 256400 | 172200 | 263300 |
| Brain Sample #7 | 131800 | 205200 | 151000 | 229000 | 163700 | 234000 |
| Brain Sample #8 | 133900 | 216900 | 159300 | 245100 | 178400 | 263100 |
| Brain Sample #9 | 66570 | 86140 | 102200 | 103500 | 55200 | 42100 |
| Brain Sample #10 | 49420 | 67220 | 63850 | 80060 | 72410 | 135000 |
| Brain Sample #11 | 54780 | 67260 | 73840 | 75280 | 124400 | 196100 |
| Brain Sample #12 | 45910 | 70890 | 62770 | 85940 | 63490 | 131100 |
| Brain Sample #13 | 49190 | 60530 | 68830 | 69930 | 65500 | 100400 |
| Brain Sample #14 | 63340 | 73470 | 86330 | 78890 | 99990 | 141900 |
| See Materials and Methods for processing of human brain specimens. |  |  |  |  |  |  |
