# Supplementary material for: Phosphorylated Dihydroceramides from Common Human Bacteria Are Recovered in Human Tissues
Source: PLoS One. 2011 Feb 11;6(2):e16771. doi: 10.1371/journal.pone.0016771 (PMC3037954; doi:10.1371/journal.pone.0016771)
Supplement: Table S3 — Ion abundances of bacterial phosphorylated dihydroceramides in lipid extracts of paired common carotid (control) and carotid atheroma samples derived from human endarterectomy samples. The individual tissue specimens were processed as described in the Materials and Methods and individual lipid extracts were evaluated by MRM-MS. Electronically integrated peaks depicted here were used to generate the summary results shown in Figure 3. (DOC) [file pone.0016771.s003.doc]

Table S3

|  |  |  |  | Transition Ion Abundances | |  |  |
| --- | --- | --- | --- | --- | --- | --- | --- |
| Carotid Sample | Category | HM SubPG DHC | HM UnPG DHC | LM SubPG DHC | LM UnPG DHC | HM PE DHC | LM PE DHC |
| 1 | Atheroma | 286500 | 111300 | 165500 | 131900 | 69870 | 71580 |
| 1 | Control | 5014000 | 309600 | 2741000 | 152200 | 247500 | 99760 |
| 2 | Atheroma | 238200 | 25350 | 103100 | 36270 | 211800 | 47410 |
| 2 | Control | 2698000 | 41940 | 1272000 | 27680 | 651600 | 393900 |
| 3 | Atheroma | 89830 | 38630 | 66480 | 37990 | 18800 | 15710 |
| 3 | Control | 20040 | 18980 | 12350 | 20510 | 75280 | 39210 |
| 4 | Atheroma | 63830 | 20400 | 30360 | 25810 | 26410 | 18880 |
| 4 | Control | 2392000 | 74180 | 1054000 | 54390 | 1731000 | 707900 |
| 5 | Atheroma | 345600 | 25080 | 149000 | 30210 | 35140 | 37230 |
| 5 | Control | 31640000 | 63990 | 14540000 | 218100 | 17270000 | 7222000 |
