# Supplementary material for: Phosphorylated Dihydroceramides from Common Human Bacteria Are Recovered in Human Tissues
Source: PLoS One. 2011 Feb 11;6(2):e16771. doi: 10.1371/journal.pone.0016771 (PMC3037954; doi:10.1371/journal.pone.0016771)
Supplement: Table S4 — Mass spectrometric calibration parameters used to quantify bacterial lipids in bacterial or human specimens. The instrument parameters for the MRM-MS analysis are listed for the 4000QTrap Instrument (ABSciex). These parameters were defined using highly purified preparations of each PDHC lipid class. (DOC) [file pone.0016771.s004.doc]

Table S4

| Qtrap instrument parameters | | |  |  |  |  |
| --- | --- | --- | --- | --- | --- | --- |
| Instrument parameter | Value | Units |  |  |  |  |
| Curtain gas pressure | 20 | psi |  |  |  |  |
| Collision gas pressure | High | pressure |  |  |  |  |
| Ion spray voltage | -4500 | V |  |  |  |  |
| Ion source temperature | 425 | ˚C |  |  |  |  |
| Ion gas 1 pressure | 35 | psi |  |  |  |  |
| Ion gas 2 pressure | 40 | psi |  |  |  |  |
| Declustering potential | -450 | V |  |  |  |  |
| CEM | 2200 | V |  |  |  |  |
|  |  |  |  |  |  |  |
| Bacterial PDHC | Q1 (*m/z*) | Q3 (*m/z*) | Time (mSec) | EP (V) | CE (V) | CXP (V) |
| HM PE DHC | 705 | 140 m/z | 150 | -10 | -52 | -20 |
| LM PE DHC | 677 | 140 m/z | 150 | -15 | -60 | -30 |
| HM UN PG DHC | 737 | 171 m/z | 150 | -10 | -52 | -20 |
| LM UN PG DHC | 709 | 171 m/z | 150 | -15 | -60 | -20 |
| HM Sub PG DHC | 960 | 171 m/z | 150 | -10 | -78 | -10 |
| LM Sub PG DHC | 932 | 171 m/z | 150 | -10 | -76 | -10 |
|  |  |  |  |  |  |  |
| EP - Entrance potential |  |  |  |  |  |  |
| CE - Collision energy |  |  |  |  |  |  |
| CXP - Collision cell exit potential | |  |  |  |  |  |
